# Correlating Fibrinogen Consumption and Profiles of Inflammatory Molecules in Human Envenomation's by *Bothrops atrox* in the Brazilian Amazon

**DOI:** 10.3389/fimmu.2020.01874

**Published:** 2020-08-18

**Authors:** Irmgardt Alicia María Wellmann, Hiochelson Najibe Santos Ibiapina, Jacqueline Almeida Gonçalves Sachett, Marco Aurélio Sartim, Iran Mendonça Silva, Sâmella Silva Oliveira, Andréa Monteiro Tarragô, Ana Maria Moura-da-Silva, Marcus Vinícius Guimarães Lacerda, Luiz Carlos de Lima Ferreira, Adriana Malheiro, Wuelton Marcelo Monteiro, Allyson Guimarães Costa

**Affiliations:** ^1^Programa de Pós-Graduação em Medicina Tropical, Universidade do Estado do Amazonas (UEA), Manaus, Brazil; ^2^Instituto de Pesquisa Clínica Carlos Borborema (IPCCB), Fundação de Medicina Tropical Dr. Heitor Vieira Dourado (FMT-HVD), Manaus, Brazil; ^3^Programa de Pós-Graduação em Imunologia Básica e Aplicada, Universidade Federal do Amazonas (UFAM), Manaus, Brazil; ^4^Programa de Pós-Graduação em Ciências Aplicadas à Hematologia, Universidade do Estado do Amazonas (UEA), Manaus, Brazil; ^5^Diretoria de Ensino e Pesquisa, Fundação Hospitalar de Hematologia e Hemoterapia do Amazonas (HEMOAM), Manaus, Brazil; ^6^Laboratório de Imunopatologia, Instituto Butantan, São Paulo, Brazil; ^7^Instituto de Pesquisas Leônidas e Maria Deane, FIOCRUZ-Amazônia, Manaus, Brazil

**Keywords:** hemostasis, immune response, *Bothrops* snakebites, inflammation-coagulation, crosstalk

## Abstract

Snakebites are considered a major public health problem worldwide. In the Amazon region of Brazil, the snake *Bothrops atrox* (*B. atrox*) is responsible for 90% of the bites. These bites may cause local and systemic signs from acute inflammatory reaction and hemostatic changes, and present common hemorrhagic disorders. These alterations occur due the action of hemostatically active and immunogenic toxins which are capable of triggering a wide range of hemostatic and inflammatory events. However, the crosstalk between coagulation disorders and inflammatory reaction still has gaps in snakebites. Thus, the goal of this study was to describe the relationship between the consumption of fibrinogen and the profile of inflammatory molecules (chemokines and cytokines) in evenomations by *B. atrox* snakebites. A prospective study was carried out with individuals who had suffered *B. atrox* snakebites and presented different levels of fibrinogen consumption (normal fibrinogen [NF] and hypofibrinogenemia [HF]). Seventeen patients with NF and 55 patients with HF were eligible for the study, in addition to 50 healthy controls (CG). The molecules CXCL-8, CCL-5, CXCL-9, CCL-2, CXCL-10, IL-6, TNF, IL-2, IL-10, IFN-γ, IL-4, and IL-17A were quantified in plasma using the CBA technique at three different times (pre-antivenom therapy [T0], 24 h [T1], and 48 h [T2] after antivenom therapy). The profile of the circulating inflammatory response is different between the groups studied, with HF patients having higher concentrations of CCL-5 and lower IFN-γ. In addition, antivenom therapy seems to have a positive effect, leading to a profile of circulating inflammatory response similar in quantification of T1 and T2 on both groups. Furthermore, these results suggest that a number of interactions of CXCL-8, CXCL-9, CCL-2, IL-6, and IFN-γ in HF patients are directly affected by fibrinogen levels, which may be related to the inflammatory response and coagulation mutual relationship induced by *B. atrox* venom. The present study is the first report on inflammation-coagulation crosstalk involving snakebite patients and supports the better understanding of envenomation's pathophysiology mechanisms and guides in the search for novel biomarkers and prospective therapies.

## Introduction

Snakebite is a neglected tropical disease and a major public health problem in developing countries worldwide. It is an important cause of morbidity and mortality, especially in areas of extreme poverty in the tropics and subtropics, such as sub-Saharan Africa, South, and Southeast Asia, Papua New Guinea, Central and Latin America (1). Approximately 421,000 cases of snakebites and 20,000 deaths occur worldwide each year (2, 3). In Brazil, specifically in the Amazon region, 57,374 notifications of snakebites were reported, resulting in incidence rate of 37.2 cases per 100,000 inhabitants/year, during the period from 2010 to 2015 (4). The *B. atrox* snake is widely distributed in the northern region of Brazil (5, 6), and is the species that causes 90% of envenomings in the region (7).

Clinical manifestations observed in victims of *Bothrops* snakebites are characterized by varied local and systemic effects, as a result of the action of biologically active toxins in the venom (2, 8). Among these effects, inflammatory and hemostatic disorders are frequently observed in patients. Envenomation's by viperid snakes often cause local and systemic bleeding (9, 10), such as gingivorrhagia, epistaxis, hematemesis, hematuria, bleeding in the uteris, and placenta (in pregnant women) (11, 12) and bleeding in the central nervous system (13–16).

Coagulopathy as a result of a snakebite is characterized by the action of hemostatically active toxins, which are capable of interfering with coagulation factors, by activating, inhibiting, or modulating platelet function, and inducing fibrinolysis. These effects can lead to blood incoagulability, which is characterized by the depletion of coagulation factors, and this depletion can be accompanied by thrombocitopenia, as well as intravascular thrombotic events. Consumption coagulopathy, thrombocytopenia and the effect of hemorraghins (toxins that act in the degradation of components of the basement membrane of vessels and with direct cytotoxic action on endothelial cells) are involved in the development of hemorrhagic events. The results consist of local and systemic bleeding disorders, hypovolemic shock, and thrombotic microangiopathy (17).

Regarding the inflammatory process, the *Bothrops* snakebite is characterized by the ability of venom toxins to directly activate inflammatory cells that are circulating or tissue located (18). It can also indirectly activate them as leukocytes recognize products of tissue damage caused by the action of venom components, known as venom-associated molecular patterns (VAMPs), and damage-associated molecular patterns (DAMPs) (19–21). In both cases, leukocyte stimulation is responsible for the production of inflammatory mediators, such as cytokines, chemokines, lipid mediators, and components of the complement system, resulting in a leukocyte infiltrate. The exacerbated inflammatory reaction enhances the tissue damage and reduces repair mechanisms, contributing to local complications from snakebites such as edema formation, necrosis, compartment syndrome, functional deficit, and amputation, as well as alterations in organs such as the kidneys and lungs (16, 20, 22, 23).

Coagulation and inflammation are highly integrated and well-balanced biological systems since systemic inflammation can lead to activation of coagulation and the components of these can modulate the inflammatory response. Deregulation components of these systems can affect this balance, resulting in a large number of diseases with different levels of severity associated with excessive inflammation and thrombosis (24). However, the crosstalk between coagulation disorders and inflammatory reaction still has gaps in snakebites. Current knowledge demonstrates that isolated *Bothrops* venom and toxins can be associated with acute inflammatory processes and coagulation disorders (25).

Patients bitten by *B. atrox* snakes in the Amazonas State, Brazil, presented the installation of a consumption coagulopathy, in which 85% of the patients showed hypofibrinogenemia (26). In addition, increased levels of soluble inflammatory mediators in patients have been demonstrated, and are associated with increased severity of the accident (27).

The aims of this study were to describe the relationship between the consumption of fibrinogen and the profile of inflammatory molecules (cytokines and chemokines) in *B. atrox* snakebite patients in the Brazilian Amazon. Our results show that inflammatory molecules showed different interactions associated with the levels of fibrinogen, which suggests crosstalk between coagulation disorders and inflammatory reactions.

## Materials and Methods

### Study Design

A prospective study was carried out with individuals who had suffered *B. atrox* snakebites and who had sought medical assistance at the *Fundação de Medicina Tropical Doutor Heitor Vieira Dourado* (FMT-HVD).

### Patients and Sampling

**Figure 1:**
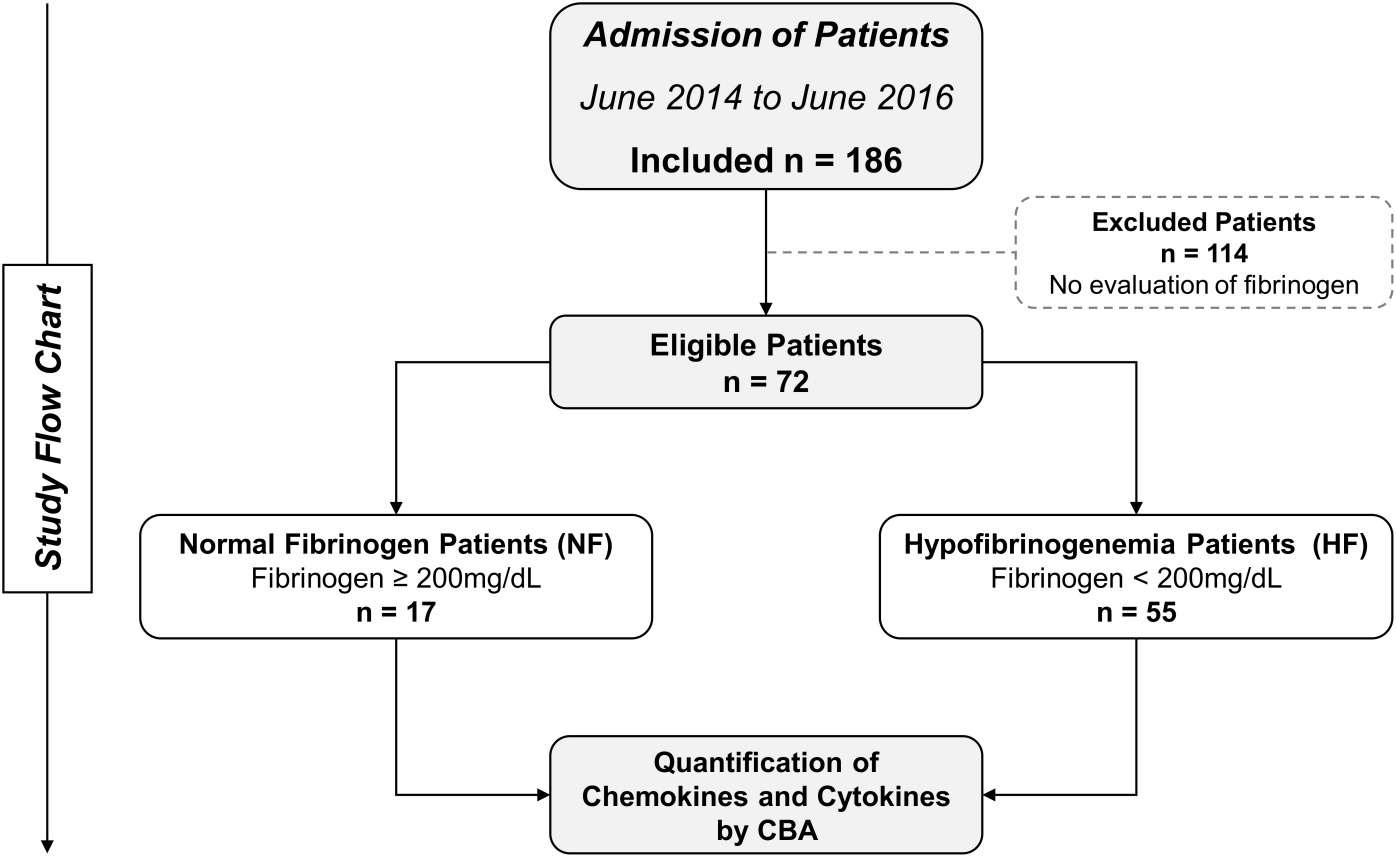
Flowchart of study. Seventy-two patients were eligible and followed up until discharge. These patients were divided into two groups: normal fibrinogen (NF) and hypofibrinogenemia (HF), according to their fibrinogen consumption.

The study population consisted of 72 individuals with clinical and laboratory diagnosis of *Bothrops* snakebite and who had sought medical assistance at FMT-HVD. Pregnant women or individuals who reported a history of chronic inflammatory disease, autoimmune diseases, or immunodeficiency were not included. *Bothrops* identification was performed by a zoologist from the research group. Patients were included, and then followed-up for 48 h, with clinical and laboratory evaluations carried out on three occasions: before antivenom therapy (T0), at 24 h (T1), and at 48 h (T2) after the administration of antivenom. Patients were classified into two subgroups (normal fibrinogen [NF], values ≥200 mg/dL; and hypofibrinogenemia [HF] <200 mg/dL) according to their results of fibrinogen consumption (Figure 1). Furthermore, blood samples from patients were subsequently sent to the Instituto Butantan (IBu) to confirm snakebite by *Bothrops* sp. using a gender-specific ELISA (28). In addition, 50 healthy individuals, of either gender and no history of snakebite, were included in the control group (CG) as a comparison parameter considering the baseline levels of cytokines and chemokines obtained at the *Fundação Hospitalar de Hematologia e Hemoterapia do Amazonas* (HEMOAM).

### Ethical Issues

This study was approved by the FMT-HVD Research Ethics Commitee (process #492.892/2013). Participants read and signed the informed consent form before enrollment. All patients were treated according to Brazilian Ministry Health protocols (29).

### Biological Sample Collection and Clinical Data

The quantification of inflammatory molecules was performed with ~4 mL of peripheral blood collected at three different times (T0, T1, and T2) by venipuncture in tubes containing EDTA (BD Vacutainer^®^ EDTA K2). In addition, for the measurement of fibrinogen in the first 24 h, peripheral blood samples were collected in tubes with sodium citrate (BD Vacutainer^®^ sodium citrate). After collection, the samples were centrifuged (1,200 g for 5 min.) for the acquisition of a plasma aliquot (500 uL) and storage in a freezer at −80°C, with a view to subsequent measurement. The clinical-epidemiological data of the patients (gender, age, previous history of snakebite, area of occurrence, severity classification of the case, and affected anatomical region) were systematically collected.

### Fibrinogen Quantification

Fibrinogen was quantified based on the Clauss method (30), using the Fibrinogen-C Kit (HemosIL^®^, Instrumental Laboratory Company, USA. Kit code: 0020301100) in an ACL TOP 300CTS coagulation analyzer (Werfen Instrumentation Laboratory, Barcelona, Spain). The test was carried out according to the manufacturer's protocol.

### Inflammatory Molecules Level Quantification by CBA

The measurement of chemokines and cytokines in patients' plasma samples was performed using the CBA (Cytometric Bead Array) Flow Cytometry technique with the BD™ CBA Human Chemokine Kit (Code: 552990, BD^®^ Biosciences, San Diego, CA, USA) and BD™ CBA Human Th1/Th2/Th17 Cytokine Kit (Code: 560484, BD^®^ Biosciences, San Diego, CA, USA) following the guidelines described by the manufacturer. The samples were acquired using a FACS Canto II Flow Cytometer (Becton, Dickinson and Company, San Jose, CA, USA) at the HEMOAM. FCAP-Array™ software (v3) was used to calculate the concentrations in pg/mL of each molecule.

### Statistical Data Analysis

The clinical data of patients and physical/electronic records, and the results of measurements of fibrinogen, cytokines, chemokines were entered into a database developed using Microsoft Excel. Statistical analyses were performed using the GraphPad Prism (v5.0) and Stata (v13.0) software. Initially, tests were performed to verify normality of data using the Shapiro-Wilk test, which showed results with non-parametric distribution. The comparisons of values between two groups of data were performed using a Mann-Whitney test, while for comparisons of variables with three or more groups, a Kruskal-Wallis test was used, followed by Dunn's *post-test*, for multiple comparisons between groups. The elaboration of networks and a demonstration of complex interactions between the fibrinogen, chemokines, and cytokines evaluated in the study were performed based on the association of these markers in each clinical group (31). Spearman's correlation test was carried out and subsequently the construction of the networks with the Cytoscape 3.7.2 software (Cytoscape Consortium San Diego, CA, USA) were done, following the recommendations and instructions described by the manufacturer. The levels of statistical significance defined in all tests were *p* < 0.05.

## Results

### Clinical and Epidemiological Baseline of the Patients

The clinical and epidemiological characteristics of the patients showed that the male gender was most common, and median age was statistically higher in *B. atrox* patients, when compared to controls. Table 1 summarizes the clinical and epidemiological characteristics of the individuals included in the study. Most *B. atrox* patients had no previous history of snakebites and bites had occurred in rural areas. The main anatomical site of the snakebite was the lower limbs and bite classification was mild occurred in *B. atrox* groups. The time between the snakebite and the antivenom therapy administration was no different in patients.

**Table 1:** Demographic and clinical characteristics of patients and individuals included in the study.

**Demographic and clinical characteristics**	**Control group**	***B. atrox*** **patients**		***p-value***
		**Normal fibrinogen**	**Hypo-fibrinogen**	
	***n* = 50**	***n* = 17**	***n* = 55**	
Gender (*n*, Male/Female)	34/16	14/3	47/8	0.248
Age (Years, median and [IQR])	30 [23–42]	45 [27–55]	39 [29–57]	**0.006**
Previous snakebite (*n*, Yes/No)	-	1/16	11/44	0.271
Occurrence zone (*n*, Rural/Urban)	-	15/2	50/5	0.665
Anatomical site of the Snakebite (*n*, Upper/Lower limb)	-	1/16	14/41	0.099
Accident Classification (*n*, Mild/Severe)	-	15/2	12/7	0.127
Time from Snakebite to Antivenom (hours, median [IQR])	-	4 [3–7]	4 [3–8]	0.591

*Values in bold show a statistically significant difference*.

### *B. atrox* Snakebite Patients With Hypofibrinogenemia (HF) Presented Similar Plasmatic Levels of Inflammatory Molecules When Compared to Those in the Normal Fibrinogen (NF) Group

**Figure 2:**
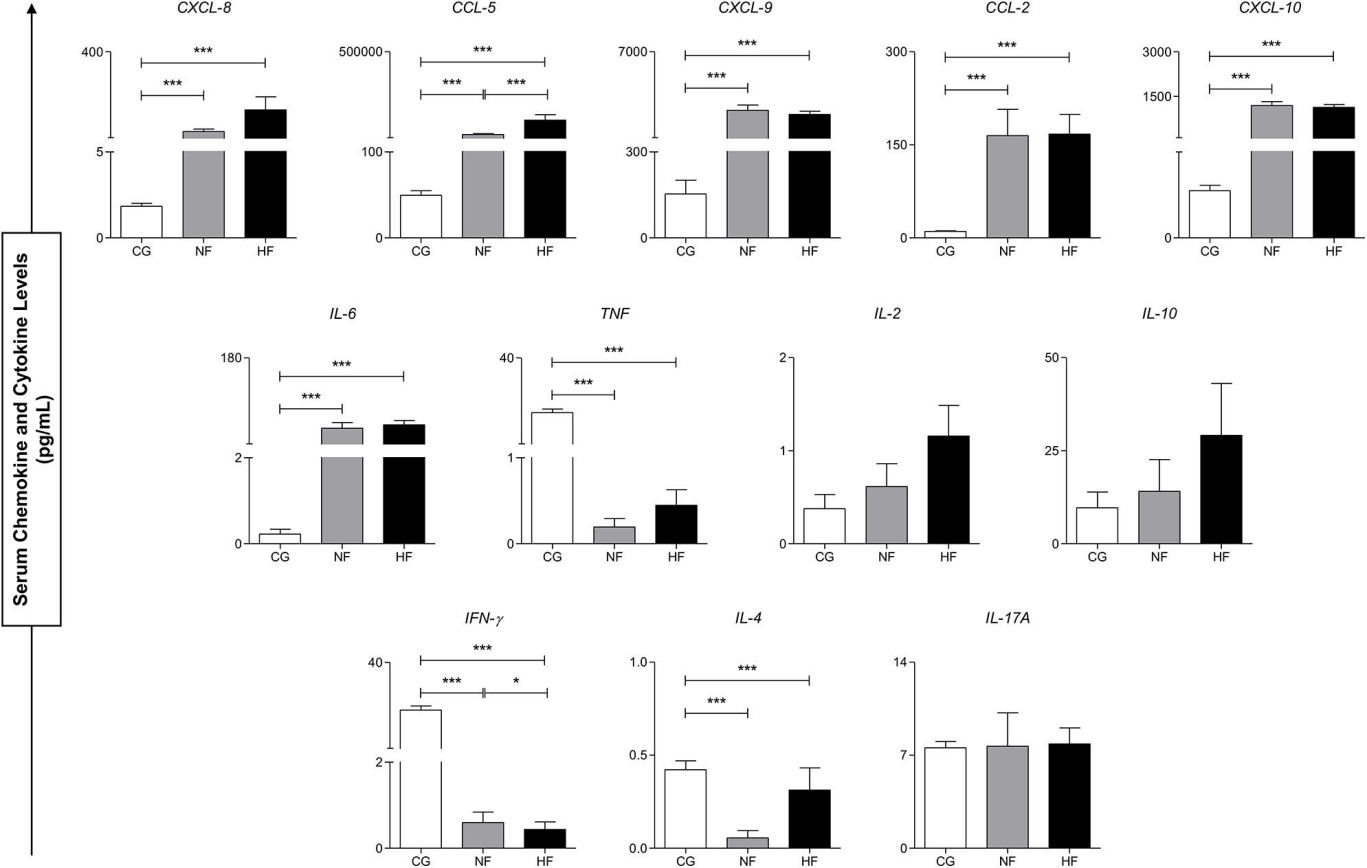
Serum concentrations of circulating molecules of control groups (CG), normal fibrinogen (NF) and hypofibrinogenemia (HF) at T0. ^*^p < 0.05; ^***^p < 0.0001.

Figure 2 demonstrates the production dynamics of inflammatory molecules in the control group (CG), *B. atrox* snakebite with normal fibrinogen (NF) and hypofibrinogenemia (HF) before antivenom administration (T0). The inflammatory molecules CXCL-8, CCL-5, CXCL-9, CCL-2 CXCL-10, and IL-6 were higher in patients with *B. atrox* snakebite (NF and HF groups) when compared to the CG group. In addition, TNF, IFN-γ, and IL-4 showed low concentrations in these patients. Furthermore, profile of the circulating inflammatory molecules is similar between the NF and HF snakebite groups, with HF patients presenting higher concentrations of CCL-5 and lower IFN-γ.

### Production Dynamics of Inflammatory Molecules in *B. atrox* Snakebite Patients With Normal Fibrinogen (NF) and Hypofibrinogenemia (HF) After Antivenom Therapy

**Figure 3:**
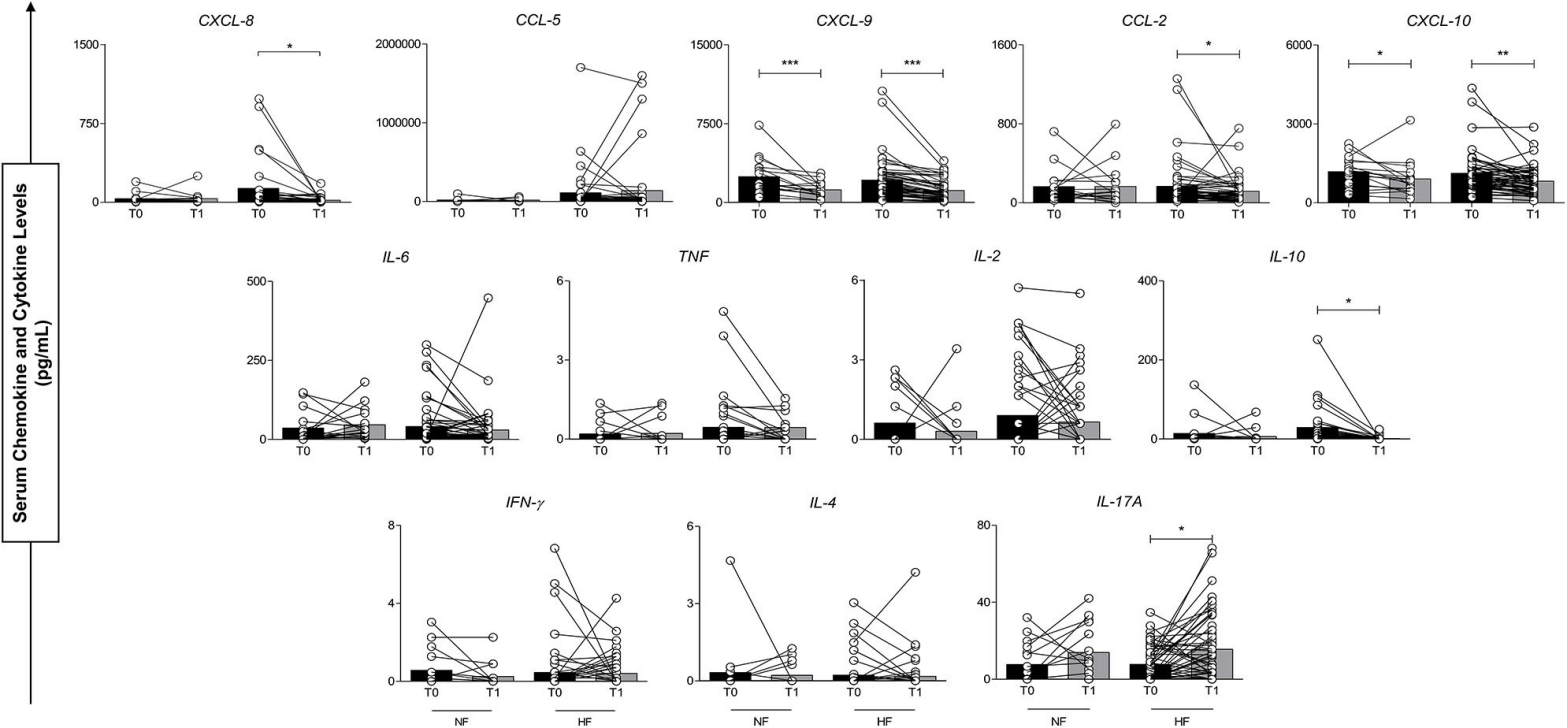
Serum concentrations of circulating molecules of groups with normal fibrinogen (NF) and hypofibrinogenemia (HF) at T0 and T1. ^*^p < 0.05; ^**^p < 0.01; ^***^p < 0.0001.

Differences in plasma concentrations of inflammatory molecules were evaluated before antivenom administration and 24 h after antivenom administration (T0 vs. T1), in the NF and HF *B. atrox* snakebite groups. This analysis shows that the elements had different behavior in the first 24 h after treatment. Figure 3, it can be noted that there was a significant decrease for CXCL-9 and CXCL-10 in both groups. CXCL-8, CCL-2, and IL-10 showed a significant decrease only in the HF *B. atrox* snakebite patients. In addition, it can be noted that IL-17A showed an upward trend in *B. atrox* groups, and this increase was only statistically significant in the HF group (Figure 3).

### Administration of Antivenom Therapy Had a Positive Effect in the Profile of Circulating Inflammatory Molecules in Follow-Up of *B. atrox* Snakebite Groups

**Figure 4:**
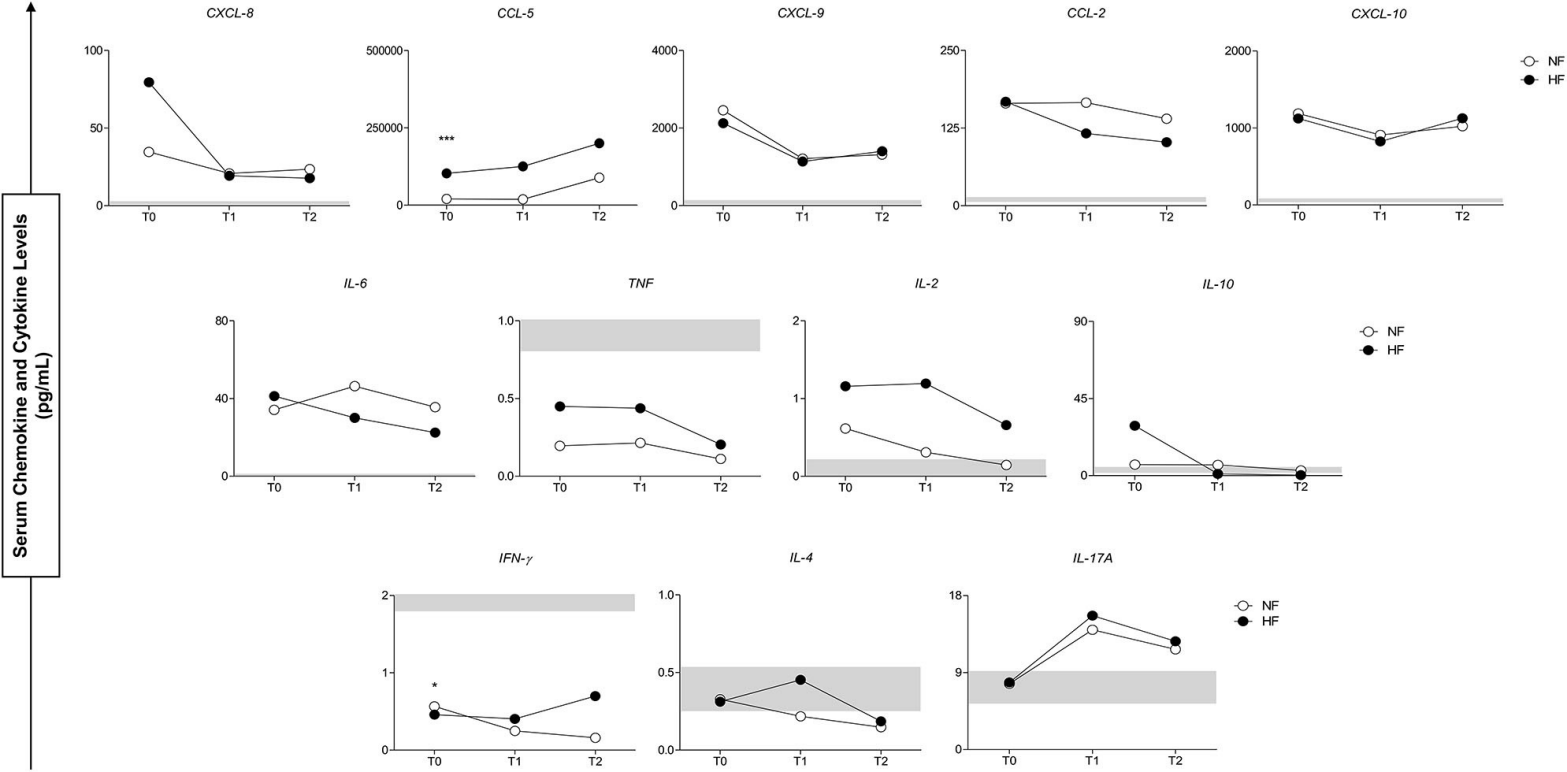
Serum concentrations of circulating molecules of groups with normal fibrinogen (NF) and hypofibrinogenemia (HF) in follow-up of study (T0–T2). At the bottom, the interquartile range (25–75) of the serum concentrations in the CG group is shown as the baseline parameter. ^*^p < 0.05; ^***^p < 0.0001.

Figure 4 shows a complementary view of the results of Figure 3, where the plasma concentration of the inflammatory molecules and the possible temporal variations (T0, T1, and T2) between groups were compared. The administration of antivenom had a positive effect and presented a similar profile of circulating inflammatory response in follow-ups of *B. atrox* snakebite groups and the levels of molecules analyzed.

### Network Correlation of Inflammatory Molecules in HF *B. atrox* Snakebite Patients Are Directly Affected by Fibrinogen Levels

**Figure 5:**
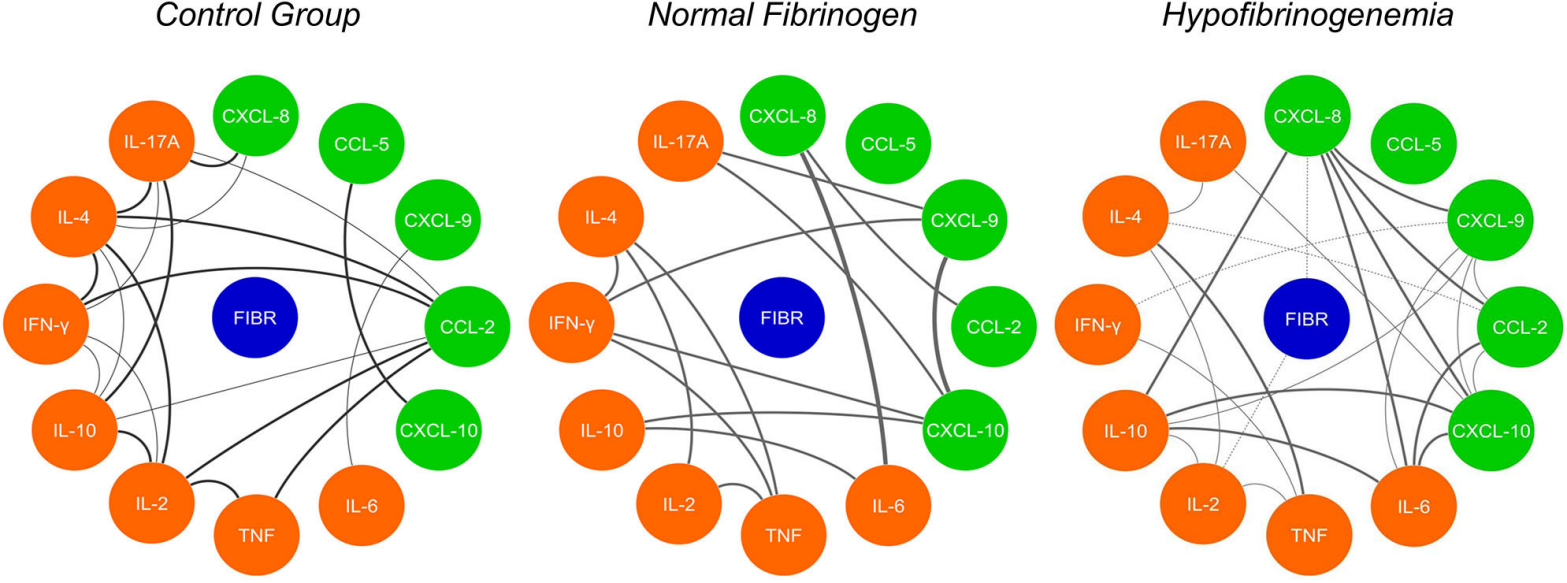
Network correlation of soluble molecules shows interactions occurring throughout the fibrinogen levels and interaction of the molecules in the control group (CG). Each group of colored nodes is used to identify chemokines (green), cytokines (orange), and fibrinogen (blue). Dashed lines indicate negative correlation and continuous lines in black, positive correlation, while thickness shows correlation strength. The correlation index (*r*) was used to categorize the correlation strength as weak (*r* ≤ 0.35), moderate (*r* ≥ 0.36–*r* ≤ 0.67), or strong (*r* ≥ 0.68).

The correlation networks between the inflammatory molecules allow us to understand the interaction between them and the mutual influence, which is exercised, thus allowing a clearer visualization of the polarization of the response profiles at the serum level, as shown in Figure 5. The CG network shows positive correlations between chemokines and cytokines, which is taken as a normal response in healthy individuals without any inflammatory process. Comparing the existing correlations in the groups of *B. atrox* snakebites patients (NF and HF), we noticed a great difference in the profile of circulating plasmatic molecules and in the number of correlations existing between them at the time of admission. Correlations in the NF *B atrox* snakebite group at T0 demonstrate an acute inflammatory response and strong and moderate correlations occur between chemokines (CXCL-8, CXCL-9, CCL-2, and CXCL-10), inflammatory cytokines (IL-6 and TNF). There is a predominant positive stimulus of the Th1 profile molecules (IFN-γ), but with the participation of the Th2 profile (IL-4) and Th17 profile with the cytokine IL-17A, which plays a stronger inflammatory role. There is also a regulatory process with the participation of the IL-10 and IL-2 molecules. While in the HF *B. atrox* snakebite group, it is possible to observe a predominance of a chemotactic process with positive and negative, moderate, and strong correlations between chemokines (CXCL-8, CXCL-9, CCL-2, and CXCL-10), both among these molecules as with cytokines (IL-10, IL-6, IFN-γ, IL-4, and IL-17A), with a more polarized profile for Th2 response (IL-4). When analyzing fibrinogen using inflammatory molecules, we observed that CXCL-8 and IL-2 show negative correlations with this hemostatic factor (Figure 5).

## Discussion

The complex composition of toxins present in the *Bothrops* venom is responsible for a varied pathological response, which is represented in the diverse clinical manifestations observed in patients. Hemostatic and inflammatory disorders can be considered important pathophysiological effects and are associated with the effects of different components of the venom on different cellular and molecular targets. Evidence of crosstalk between coagulation and inflammation can lead to an enhancement of the effects and to greater damage (25). Thus, the present clinical study shows for the first time the association between inflammation and coagulation in patients who were victims of *B. atrox* snakebites.

The chosen hemostatic parameter was the concentration of fibrinogen. Fibrinogen is an acute phase protein, produced by the liver, and its serum concentration may increase in inflammatory and infectious conditions which are associated with vascular damage. In addition, this product of the coagulation cascade was identified as a significant risk factor and as an inflammatory process modulator in various pathological conditions, thus showing their participation in both coagulation and inflammation. Furthermore, the upregulation of the acute phase response proteins is mediated by IL-6 (32–35). *Bothrops* venoms are composed of hemostatically active molecules where, among them, we have pro-coagulant toxins capable of activating coagulation factors and thrombin-like toxins, which are capable of directly cleaving fibrinogen into fibrin. Among the toxins isolated from the venom of *B. atrox*, serine proteinases with thrombin-like activity and metalloproteases able to activate factors II, V, X, XIII, and VIII have already been described (36–40). The intravascular action of these toxins contributes to the formation of fibrin and the consequent consumption of fibrinogen. In addition, *B. atrox* venom toxins present fibrin(ogen)olytic and thrombolytic activity which also contribute to coagulopathy (41). Among the hemostatic changes observed in patients from *B. atrox* snakebites, blood incoagulability is accompanied by low plasma levels of fibrinogen and increased levels of fibrin/fibrinogen degradation products, which characterize an intravascular pro-coagulant effect (10, 42). Our data show that 76.4% of snakebite patients have hypofibrinogenemia, which is close to values found in a previous study (26).

The action of the *B. atrox* venom (BaV) or isolated toxins are responsible for the activation of the inflammatory response by mechanisms that involve direct leukocyte activation or signaling by VAMPs or fragments of cell damage or hydrolysis of extracellular components (43–45). As a consequence of this leukocyte stimulation, migration of neutrophils, monocytes, and macrophages to the lesion site is observed, as well as the synthesis and release of several inflammatory mediators, such as chemokines (CXCL-8, CXCL-1, and CXCL-2), components of the complement system (C1q, C3a, C4a, and C5a), cytokines (IL-12p70, TNF-α, IL-1α, IL-1β, IL-6, IL-10, and INF-γ) and lipid mediators (PGE2, LTB4, and CysLeucotrienes) (6, 43, 44, 46, 47). In the present study, we observed a significant increase in CXCL-8, CCL-5, CXCL-9, CCL-2, CXCL-10, and IL-6 among the groups exposed to the venom (NF and HF), previously to antivenom therapy (T0), when compared to the control group (CG). In addition, decreased concentrations of TNF and IFN-γ were observed. These findings demonstrate a high chemotactic and inflammatory response to exposure to venom in patients and corroborate data previously described in *B. atrox* snakebites, which shows a plasma increase in inflammatory mediators CXCL-8, CCL-5, CXCL-9, CCL-2, CXCL10, IL-6, TNF-α, IL-1β, and IL-10 (27). We observed in our study a low production of INF-γ, which could be associated with the main cell type producing this molecule (NK cells). This would not be activated by BaV during the first 8 h and would end up not constituting and participating in this inflammatory process (6).

The relationship between coagulation and inflammation in the pathogenesis of vascular diseases has become a pathophysiological mechanism of great focus in basic and clinical research. The elucidation of the mechanisms involved in this relationship has helped in the development of new therapeutic strategies, as well as in the diagnosis of pathologies involving the inflammation/coagulation axis through the discovery of biomarkers (48).

In regards to the aspects in which inflammation induces coagulation changes, the inflammatory response is capable of modulating different hemostasis events such as: (i) activation of coagulation due the increased expression of intravascular tissue factor (Factor III), (ii) activation of platelets and thrombus formation through thrombo-inflammation, (iii) decrease in the expression of endogenous anticoagulants such as anti-thrombin, and (iv) reduction of fibrinolytic function. These events induce consumption coagulopathy, which is responsible for the installation of thrombotic and hemorrhagic conditions (24, 49). The present study shows that patients of *B. atrox* snakebites presented increased levels of mediators CXCL-8, CCL-5, CXCL-9, CCL-2, CXCL-10, and IL-6. These mediators have been described to be associated with thrombotic pathologies, and act in the modulation of platelet function, expression of coagulation factors and fibrinolysis (50–53).

Among the mechanisms associated with the consumption of fibrinogen, the tissue factor (TF) plays an important role. TF is a transmembrane glycoprotein, in which the extracellular domain interacts with Factor VIIa, forming the extrinsic tenase complex (Factor III/Factor VIIa), capable of activating factor X and triggering coagulation (54). The intravascular expression of TF by endothelial cells and monocytes is induced by different stimuli, since pro-inflammatory molecules IL-6, CCL-2, and CXCL-8 are described as inducing increased TF expression (55, 56). *Bothrops* snake venoms and its isolated toxins have already been described as being responsible for inducing TF expression, which is associated with pro-inflammatory events induced by the venom and its components (57–60). Recently, the proinflammatory action of a type-C lectin isolated from *Bothrops jararacussu* venom was demonstrated inducing a monocyte pro-coagulant character by increasing TF expression (61). Interestingly, a recent study by our research group also found that patients who were victims of *B. atrox* envenoming had increased levels of TF in the circulation and was associated with systemic bleeding and blood incoagulability (unpublished data).

When comparing the levels of mediators between the NF and HF groups, the chemokine CCL-5 and cytokine IFN-γ were elevated and decreased, respectively, in the group of patients with fibrinogen consumption. Chemokine CCL-5 is a chemoattractant for monocytes, produced mainly by activated CD8^+^ T cells. Its role in the coagulation process involves the modulation of different pathways, and studies show that increased levels of the chemokine in patients with ischemic myocardial pathologies are associated with the formation of atherosclerotic plaques by the process of thromboinflammation (53). The expression of TF in this mechanism is associated with the CCL-5 pathway, since studies show that the activation of CCR-5 (CCL-5 receptors) are responsible for the expression of TF (62), as well as activated CD8 + T cells which also promote expression of TF in monocytes (63). Another function is related to the ability of CCL-5 to induce platelet degranulation and aggregation. These activated platelets serve as substrates for the formation of coagulation complexes and clotting activation, culminating in the consumption of fibrinogen and leading to the formation of intravascular thrombus (64).

Previously it has been shown that the levels of circulating soluble inflammatory molecules are higher before the administration of the antivenom, demonstrating an acute response to snakebites of the genus *Bothrops* (27) which agrees with our results which allowed us to observe the drop in levels of CXCL-9 and CXCL-10 in both evaluated groups. IL-10, CCL-2, and CXCL-8 showed a significant decrease only in the HF group. In addition, IL-17A showed an upward trend in both groups, this increase was statistically significant only in the HF group. With regard to this cytokine, we emphasize that IL-17 is a pro-inflammatory molecule that activates macrophages, fibroblasts, and stromal cells, including the expression of ICAM-1 and cytokine secretion (IL-6, CXCL-8, IL-11, factor granulocyte colony stimulator [G-CSF]), prostaglandin E2, and nitric oxide) (65–67). Ding et al. reported that IL-17A promotes the pathogenesis of deep vein thrombosis (DVT), improving platelet activation and aggregation, neutrophil infiltration, and activation of endothelial cells in murine models (68).

The correlation network analyses between the molecules evaluated in the present study show that, in the group of patients with hypofibrinogenemia, it was possible to observe a predominance of a chemoattractive profile, with correlations between chemokines CXCL-8, CXCL-9, CCL-2, and CXCL-10. Leukocyte chemoattraction has an important relevance in the pathogenesis of thrombotic events. Circulating cells are recruited to the site of thrombus formation through interactions with platelets and endothelial cells, and can be induced to express TF and release pro-inflammatory and pro-coagulant molecules, which influence various aspects of thrombus formation, including activation and platelet adhesion and activation of intrinsic and extrinsic coagulation pathways (69). Furthermore, we observed that the mediators CXCL-8 and IL-2 have a correlation with the consumption of fibrinogen, both of which have already been described because they are capable of inducing an intravascular coagulation process (70, 71).

In addition to the ability of inflammation to induce coagulation, the relationship is bidirectional, so that coagulation is also capable of modulating inflammatory activity. In this case, several agents are responsible for this action as coagulation factors and products of the action of these factors (48). Among the mechanisms, coagulation factors with proteolytic function are responsible for interacting with specific cells through their receptors to induce the activation of signaling pathways. Protease-Activated Receptors (PARs), expressed in endothelial cells, mononuclear cells, platelets, and others, have an important function because they are targets of the action of thrombin, TF/VIIa factor complex and Xa factor. The production of cytokines IL-6, IL-2, CXCL-5, CXCL-8, CCL-2, TNF-α, IL-1β, and IFN-γ by these coagulation factors through activation of PARs have already been reported (72–75). Therefore, it is important to consider the participation of factors Xa and thrombin in the inflammatory process observed in our *B. atrox* snakebite patients, since activators of factor X and prothrombin are present in the BaV (38, 39). The increase in CCL-5 levels in patients who presented hypofibrinogenemia may be associated with a possible action of the generated thrombin and FXa, since these factors are responsible for the expression of this chemokine in fibroblasts, platelets, and endothelial cells (76–78). Still, the chemotactic response in HF patients associated with the CXCL-8 and IL-2 correlation to fibrinogen consumption could be associated with thrombin activity, which is capable of inducing leukocyte chemotaxis (79).

Another mechanism which involves the ability of coagulation to modulate inflammation is the action of fibrinogen and its degradation products. The cleavage of fibrinogen by thrombin or by thrombin-like toxins can generate fibrin, which forms the polymer that comprises the clot. This cleavage produces fibrin peptides, such as fibrinopeptide B, which can act as chemoattractions for leukocytes and, thus, independently modulate inflammatory responses (32). It is important to note that these pro-inflammatory functions are a product of fibrin/fibrinogen signaling through binding sites that do not overlap with those involved in the coagulation cascade. The integrin receptor CD11b/CD18 (also called Mac-1, complement receptor 3 or αMβ2) is a representative example. This receptor is expressed by leukocytes of the innate immune system, mainly circulating monocytes, tissue-specific macrophages, and microglia residing in the central nervous system (CNS). Fibrin/fibrinogen signaling through CD11b/CD18 has been shown to activate pro-inflammatory pathways, such as NF-κB, which results in the local production of inflammatory cytokines, such as TNF-α and IL-1β (32). Studies show that fibrinogen and fibrin are able to induce the production of IL-6 and TNF-α in blood mononuclear cells (80).

The study had some limitations related to the sample size of patients with normal fibrinogen dosage, quantification of other factors involved in the coagulation cascade (e.g., Tissue Factor) and patients' venom levels were not measured. However, we would like to emphasize that the results presented here help to understand this type of systemic complication; one which is common in the field of snakebites and of great clinical importance.

## Conclusion

**Figure 6:**
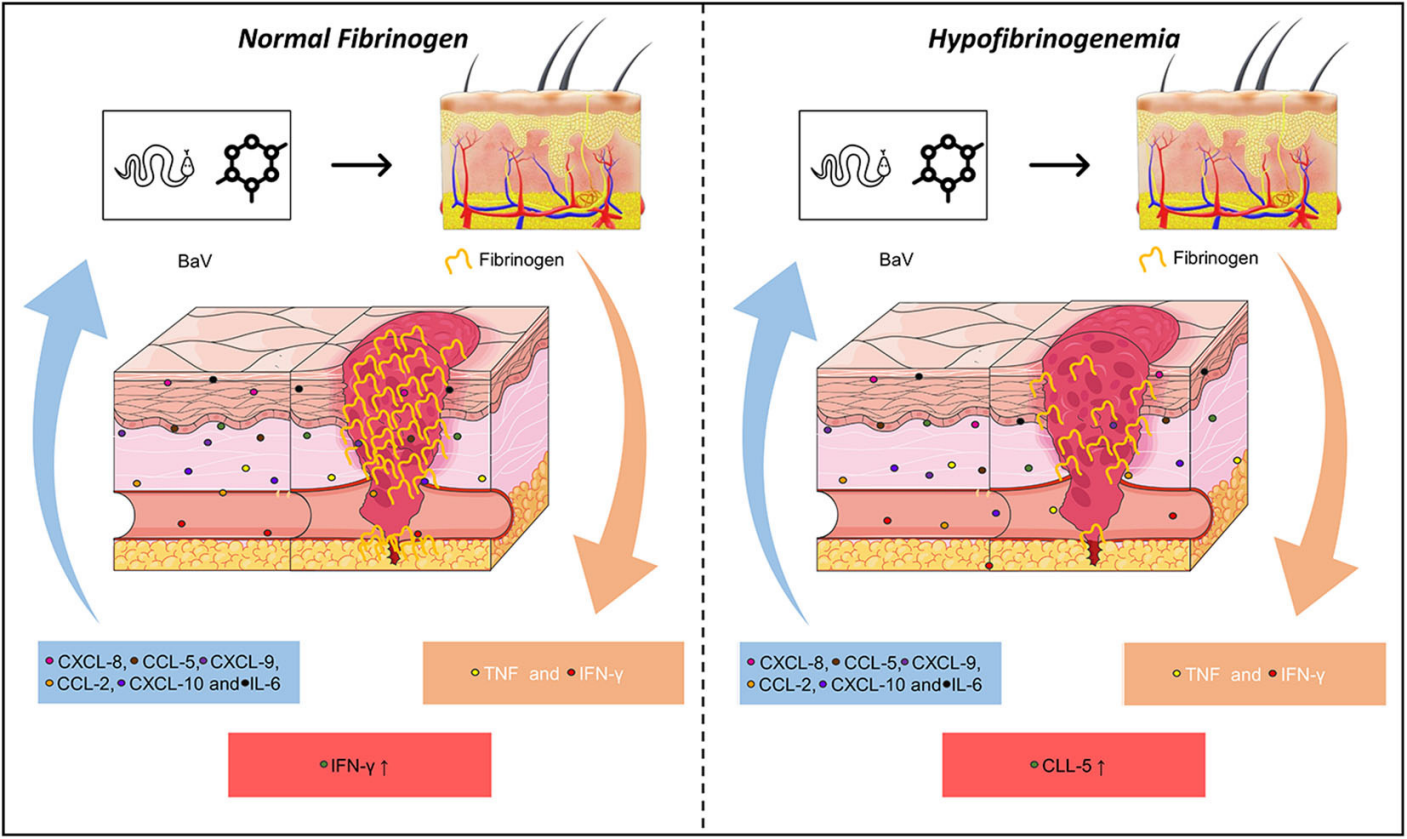
Correlating fibrinogen consumption and profiles of inflammatory molecules in *Bothrops atrox* snakebite patients. Schematic presentation of inflammation-coagulation crosstalk in patients with normal fibrinogen (NF) and hypofibrinogenemia (HF).

Our results suggest that number of interactions of CXCL-8, CXCL-9, CCL-2, IL-6, and IFN-γ in HF patients are directly affected by fibrinogen levels, which may be related to the inflammatory response and coagulation mutual relationship induced by *B. atrox* venom (Figure 6). In addition, CXCL-8 and IL-2 in HF *B. atrox* snakebite patients may be associated with the inflammation-coagulation axis. Furthermore, the hemostatic complication that occurs in patients suffering from *Bothrops* snakebites is multifactorial and the increase in this inflammatory response was reflected in the high concentrations of IL-6, IL-17A, and chemoattractive profile, which is possibly influenced by the coagulation factors activated by the venom. As BaV stimulates inflammation and activation of hemostatic factors, and these in turn contribute to the development of the inflammatory response, we have the result of a cycle in which increased vascular permeability, hypofibrinogenemia, bleeding, and subsequent morbidity occurs in patients. The present study is the first report on inflammation-coagulation crosstalk involving *B. atrox* snakebite patients, supporting the better understanding of envenomation's pathophysiology mechanisms and guides the search for novel biomarkers with therapeutic perspectives.

## Data Availability Statement

All datasets generated for this study are included in the article/supplementary material.

## Ethics Statement

The studies involving human participants were reviewed and approved by the Research Ethics Committee at the FMT-HVD (approval number 492.892/2013). Patients were treated according to the recommendations of Brazilian Health Ministry. The patients/participants provided their written informed consent to participate in this study.

## Author Contributions

IW, AC, and WM designed and performed the experiments, analyzed data, and wrote the manuscript. IW and AC analyzed data. HI and AC performed the experiments. WM, JS, MS, and AMM revised the manuscript. IW, HI, AC, WM, ML, LF, AM, AT, JS, IS, and SO conceived and supervised the project, designed the experiments, interpreted the data, wrote, and revised the manuscript. All authors read and approved the final manuscript.

## Conflict of Interest

The authors declare that the research was conducted in the absence of any commercial or financial relationships that could be construed as a potential conflict of interest.
